# The effects of endoscopic vacuum therapy for non-operative treatment of anastomotic leakage on oncological outcomes in rectal cancer patients

**DOI:** 10.1007/s00423-025-03672-1

**Published:** 2025-03-27

**Authors:** Catherine Kollmann, Beata Kusnezov, Lars Kollmann, Jasmin Schmitt, Christoph-Thomas Germer, Johan F. Lock, Sven Flemming

**Affiliations:** Department of General, Visceral, Transplant, Vascular and Paediatric Surgery, University Hospital Würzburg, Oberdürrbacherstrasse 6, 97080 Würzburg, Germany

**Keywords:** Rectal cancer, Endoscopic vacuum therapy, Anastomotic leakage, Oncological outcome

## Abstract

**Purpose:**

Rectal resection has remained the cornerstone in curative treatment of rectal cancer. This however, implies the risk of anastomotic leakage leading to morbidity, mortality and potentially disease progression. Endoscopic vacuum therapy (EVT) has emerged as a promising tool in leakage therapy in order to avoid reoperation and Hartman resection. However, its clinical efficacy and its potential effect on oncological outcomes still requires further research.

**Methods:**

In this retrospective single-centre cohort study, we analysed all consecutive patients undergoing rectal resection for rectal cancer during 2012–2021. The incidence and management of anastomotic leakage and its effects on long-term oncological outcomes were analysed.

**Results:**

A total of 334 patients underwent rectal resection of whom 47 patients (14.1%) developed postoperative anastomotic leakage. Non-operative leakage treatment (NOLT) was successful in in 76.9% of which EVT was the most efficient (90.0% success) while reoperation was successful in 52.4% (*p* = 0.073). The more frequent application of EVT increased the NOLT rate from 48.3 to 66.7% during the observation period (*p* = 0.176). Concerning long-term outcomes, no differences in disease-free survival (*p* = 0.657) nor patient survival (*p* = 0.295) could be determined.

**Conclusion:**

EVT is an effective treatment option for anastomotic leakage after rectal resection. EVT enables NOLT in the majority of cases. However, there might be no impact on oncological outcomes.

**Supplementary Information:**

The online version contains supplementary material available at 10.1007/s00423-025-03672-1.

## Introduction

Rectal cancer remains a significant global health burden, in terms of both incidence, morbidity and mortality [1–3]. Treatment modalities for rectal cancer are complex, encompassing chemotherapy, radiation therapy, targeted therapy, immunotherapy and surgery, depending on tumour stage and localization [4–7]. Until today, rectal resection constitutes a cornerstone in curative therapy of rectal cancer [8, 9].

Anastomotic leakage (AL) has remained the most relevant postoperative complication, which can lead to severe morbidity, increased rates of long-term ostomy and increased mortality [10–16]. Studies have estimated the incidence of AL between 2.5 and 19.0% depending on various patient and surgical risk factors [10–20]. These risk factors predisposing AL include e.g. male gender, obesity, advanced tumour stage, or low level of anastomosis [11, 15–17, 20]. In addition, AL is associated with significant healthcare costs due to prolonged hospital stay and necessary interventions or reoperations [12, 14, 18, 21].

Successful management of AL is crucial and various therapeutic options are available, ranging from non-operative leakage treatment (NOLT) with antibiotics and drainage up to reoperation [16, 22]. Endoscopic vacuum therapy (EVT) has emerged as a new method for gastrointestinal leakage therapy. EVT involves the endoscopic placement of a sponge into the leakage site or the leakage cavity thus applying negative pressure wound therapy. Negative pressure promotes exudate and bacterial clearance as well as granulation tissue formation [23]. Multiple reports have shown encouraging results of EVT not only after colorectal resections but also after upper gastrointestinal and bariatric surgeries with success rates of 79.7–100% [19, 24–30].

The importance of sufficient therapeutic strategies for AL is emphasized by mounting evidence that patients who experience AL could have decreased long-term survival and an increased risk of local or distant tumour recurrence [11, 20, 31–34]. A potential explanation could be the negative effect of AL on adjuvant chemotherapy [20, 32].

Despite the increasing adoption of EVT, its impact on NOLT of AL and oncological outcomes has remained undefined. The aim of the present study was to provide new insights concerning these issues based on a large patient cohort.

## Materials and methods

### Study design and population

We retrospectively analysed patients undergoing rectal resection at the University Hospital Würzburg during January 2012 and December 2021. Inclusion criteria were rectal resection (high or low anterior resection) for rectal cancer with the creation of a primary intestinal anastomosis and age ≥ 18 years. Exclusion criteria were revisional surgery for chronic anastomotic leakage prior to index surgery in our institution. In summary, 334 patients were included in the analysis.

Demographical and clinical data was collected retrospectively. Apprehended patient characteristics included age, sex, BMI and pre-existing conditions while clinical data included surgical details, preoperative laboratory results and postoperative complications. The primary endpoints were success of AL treatment and overall survival (OS) of patients with rectal cancer. Secondary endpoints were hospital length of stay (LOS), Comprehensive Complication Index (CCI) [35], complication grading according to the Clavien-Dindo Classification [35], disease-free survival (DFS) and details about adjuvant therapy. To assess the impact of AL on the clinical management of patients, Rahbari grading was applied: Grade A - AL results in no change in patient’s management, Grade B - AL requires active therapeutic intervention but is manageable without relaparotomy, Grade C - AL requires relaparotomy [36, 37].

### Ethics approval

#### Ethical approval

was given by the Ethics Committee of the University of Würzburg.

### Surgical procedures

Patients received open or minimal-invasive rectal resections by either high or low anterior resection with a primary intestinal anastomosis. Intestinal anastomosis was performed by either 25–31 mm circular stapler (Covidien, Dublin Ireland; Frankenman, Kiel, Germany; Ethicon, Hamburg, Germany) or handsewn sutures. A protective ostomy was optional depending on the type of surgery.

### Leakage management

AL was diagnosed by endoscopy or computer tomography. If endoscopy revealed an apparent anastomotic defect, AL was determined to be macroinsufficient. If no defect was visible during endoscopy, but the anastomosis fibrin covered and CT showed signs of insufficiency (free air, perianastomotic collection) a microinsufficiency was diagnosed. Antiinfective treatment was started in patients that showed signs of systemic infection. If clinically necessary, further treatment was initiated. This included transanal catheter or EVT. If the anastomosis was completely necrotic or sepsis with generalised peritonitis occurred, reoperation was indicated. Success was defined as achieving anastomotic healing without any second line therapies while preserving the anastomosis. Reoperation with Hartmann procedure was determined as unsuccessful AL treatment.

### Endoscopic vacuum-therapy

The local EVT technique has been previously described in detail [25, 26, 38]. EVT for AL after rectal resection was implemented in 2017 in our institution.

### Statistical analysis

Statistical analysis was performed using SPSS Statistics 29 (IBM Corporation, Armonk, NY, USA). Contingencies were calculated using Pearson-Chi-squared test or Fisher’s exact test depending on observed frequencies. Parametric and non-parametric data were analysed using unpaired t-test or Mann-Whitney-U test respectively or ANOVA plus Welch correction depending on sample size and distribution. Descriptive data are presented as averages with 95% confidence intervals or absolute and relative frequencies. Survival rates are displayed and calculated using Kaplan-Meier analyses and Log-Rank (Mantel-Cox) Regression. Statistical significance was assumed for *p* < 0.05.

## Results

### Patient characteristics

A total of 334 patients with primary anastomosis after rectal resection were available for analysis. Detailed patient characteristics are provided in Table 1. AL was observed in 47 patients (14.1%) in total. Patient characteristics that correlated significantly with the development of AL included male sex (AL: 76.6/23.4% male/female vs. no AL: 60.3/39.7% male/female; *p* = 0.022), higher BMI (27.5 vs. 26.0; *p* = 0.031), diabetes mellitus (21.3% vs. 10.5%; *p* = 0.036), leukaemia (6.4% vs. 0.0%, *p* = 0.003), and anticoagulation (non-vitamin K antagonist anticoagulant/NOAC: 8.2% vs. 2.8%, Marcumar/Warfarin: 4.3% vs. 2.8%, other: 2.1% vs. 0.0%; *p* = 0.042).

**Table 1:** Patient characteristics

	Total (*n* = 334)	No anastomotic leakage (*n* = 287)	Anastomotic leakage (*n* = 47)	*P* value
**Sex** *n (%)*				***0.022***
Male	209 (62.6)	173 (60.3)	36 (76.6)	
Female	125 (37.4)	114 (39.7)	11 (23.4)	
**Age** *[y] avg (95%CI)*	63.4 (62.2–64.6)	63.4 (62.1–64.7)	63.4 (60.6–66.2)	*0.997*
**BMI** *[kg/m* ^*2*^ *] avg (95%CI)*	26.2 (25.8–26.7)	26.0 (25.5–26.5)	27.5 (26.0–29.0)	***0.031***
**Smoking** *n (%)*	47 (14.6)	37 (13.3)	10 (22.2)	*0.273*
Data missing	11	9	2	
**Alcohol consumption** *n (%)*	41 (13.3)	39 (14.6)	2 (4.8)	*0.186*
Data missing	25	20	5	
**ASA classification** *n (%)*				*0.474*
ASA < 3	232 (69.5)	200 (69.7)	32 (68.1)	
ASA ≥ 3	102 (30.5)	87 (30.3)	15 (31.9)	
**Charlson Comorbidity Index** *avg (95%CI)*	5.3 (5.0-5.5)	5.3 (5.0-5.5)	5.3 (4.7-6.0)	*0.862*
**Cardiovascular diseases** *n (%)*				
Myocardial infarction	31 (9.3)	23 (8.0)	8 (17.0)	*0.051*
Congestive heart failure	8 (2.4)	5 (1.7)	3 (6.4)	*0.088*
Peripheral vascular disease	6 (1.8)	5 (1.7)	1 (2.1)	*0.600*
Cerebrovascular disease	16 (4.8)	14 (4.9)	2 (4.3)	*0.603*
**Other comorbidities** *n (%)*				
Diabetes mellitus	40 (12.0)	30 (10.5)	10 (21.3)	***0.036***
Chronic pulmonary disease	27 (8.1)	22 (7.7)	5 (10.6)	*0.325*
Liver disease	10 (3.0)	7 (2.4)	3 (6.4)	*0.154*
CKD stage ≥ 3B	12 (3.6)	9 (3.1)	3 (6.4)	*0.230*
Leukemia	3 (0.9)	0 (0.0)	3 (6.4)	***0.003***
Liver metastases at time of surgery	41 (12.3)	40 (13.9)	1 (2.1)	***0.011***
**Preoperative hemoglobin** *avg (95%CI)*	13.0 (12.8–13.1)	13.0 (12.8–13.2)	12.8 (12.3–13.3)	*0.394*
**Preoperative albumin** *avg (95%CI)*	4.4 (4.3–4.4)	4.4 (4.3–4.4)	4.3 (4.2–4.4)	*0.367*
**Anticoagulation** *n (%)*				***0.042***
NOAC	11 (3.3)	8 (2.8)	3 (6.4)	
Marcumar/Warfarin	10 (3.0)	8 (2.8)	2 (4.3)	
Other anticoagulation	1 (0.3)	0 (0.0)	1 (2.1)	
No anticoagulation	312 (93.4)	271 (94.4)	41 (87.2)	
**Immunosuppression** *n (%)*				*0.466*
Corticosteroids	5 (1.5)	5 (1.7)	0 (0.0)	
No immunosuppressors	329 (98.5)	282 (98.3)	47 (100.0)	

BMI = body mass index; CKD = chronic kidney disease

### Surgical details

The surgical details are provided in Table 2. All patients received scheduled operations, there were no emergency surgeries. Patients who developed AL had received a combined stapler and hand-sewn anastomotic technique more often (19.1% vs. 8.4%) and fewer stapled anastomoses (68.1% vs. 84.3%; *p* = 0.023). Patients that developed an AL presented lower rates of intraoperative tests for leakage (73.9% vs. 86.1%; *p* = 0.034) and higher rates of oversewing of the anastomosis (21.7% vs. 8.9%; *p* = 0.014). Protective ostomy was existent in 38/47 patients that developed an AL compared to 223/287 patients without an AL which did not influence the rate of AL (80.9% vs. 77.7%; *p* = 0.394).

**Table 2:** Surgical details

	Total (*n* = 334)	No anastomotic leakage (*n* = 287)	Anastomotic leakage (*n* = 47)	*P* value
**Type of surgery** *n (%)*				*0.442*
High anterior resection	85 (25.4)	74 (25.8)	11 (23.4)	
Low anterior resection	249 (74.6)	213 (74.2)	36 (76.6)	
**Surgical approach** *n (%)*				*0.392*
Robotic	40 (12.0)	35 (12.2)	5 (10.6)	
Laparoscopic	95 (28.4)	86 (30.0)	9 (19.1)	
Open	179 (53.6)	150 (52.3)	29 (61.7)	
Conversion to open	20 (6.0)	16 (5.6)	4 (8.5)	
**Prior abdominal surgery** *n (%)*	149 (44.6)	129 (44.9)	20 (42.6)	*0.443*
**Duration of surgery** *[min] avg (95%CI)*	228.0 (219.7-236.4)	228.4 (219.3-237.5)	225.6 (203.5-247.8)	*0.820*
**Type of anastomosis** *n (%)*				*0.590*
Colorectal	311 (93.1)	267 (93.0)	44 (93.6)	
Coloanal	23 (6.9)	20 (7.0)	3 (6.4)	
**Anastomotic configuration** *n (%)*				*0.373*
Side-to-end	135 (40.4)	114 (39.7)	21 (44.7)	
End-to-end	84 (25.1)	70 (24.4)	14 (29.8)	
Pouch	115 (34.4)	103 (35.9)	12 (25.5)	
**Technique of anastomosis** *n (%)*				***0.023***
Stapler	274 (82.0)	242 (84.3)	32 (68.1)	
Hand-sewn	27 (8.1)	21 (7.3)	6 (12.8)	
Stapler and Hand-sewn	33 (9.9)	24 (8.4)	9 (19.1)	
**Test for leakage** *n (%)*	276 (82.6)	242 (86.1)	34 (73.9)	***0.034***
Data missing	7	6	1	
**Oversewing of anastomosis** *n (%)*	35 (10.5)	25 (8.9)	10 (21.7)	***0.014***
Data missing	7	6	1	
**Protective ostomy** *n (%)*	261 (78.1)	223 (77.7)	38 (80.9)	*0.394*
**Prophylactic pelvic drainage** *n (%)*	285 (85.3)	245 (85.4)	40 (85.1)	*0.555*

### Oncological data

Oncological characteristics are presented in Table 3. Most patients received neoadjuvant radio- and/or chemotherapy (212 patients, 63.5%). The most frequent pathological tumour stages were UICC I (93 patients, 27.8%), UICC II (71 patients, 21.3%) and UICC III (91 patients, 27.2%). Most patients had T2 or T3 tumours (T2: 86 patients, 25.7%; T3: 159 patients, 47.6%), no lymph node involvement (211 patients, 63.2%) and no distant metastases (280 patients, 83.8%). R0 resection was achieved in 325 patients (97.3%). There were no statistically significant differences in pre- or intraoperative oncological characteristics between patients that developed an AL and patients without leakage. Especially neoadjuvant therapy did not correlate with the development of AL (*p* = 0.861).

**Table 3:** Oncological characteristics for rectal cancer patients

	Total (*n* = 334)	No anastomotic leakage (*n* = 287)	Anastomotic leakage (*n* = 47)	*P* value
**Neoadjuvant therapy** *n (%)*				*0.861*
Radiotherapy	14 (4.2)	12 (4.2)	2 (4.3)	
Chemotherapy	15 (4.5)	14 (4.9)	1 (2.1)	
Radiochemotherapy	183 (54.8)	156 (54.4)	27 (57.4)	
No neoadjuvant therapy	122 (36.5)	105 (36.6)	17 (36.2)	
**Tumour category** *n (%)*				*0.352*
T0	28 (8.4)	23 (8.0)	5 (10.6)	
Tis	1 (0.3)	1 (0.4)	0 (0.0)	
T1	40 (12.0)	38 (13.2)	2 (4.3)	
T2	86 (25.7)	70 (24.4)	16 (34.0)	
T3	159 (47.6)	139 (48.4)	20 (42.6)	
T4	20 (6.0)	16 (5.5)	4 (8.6)	
**Nodal status** *n (%)*				*0.613*
N0	211 (63.2)	184 (64.1)	27 (57.4)	
N1	79 (23.7)	67 (23.4)	12 (25.5)	
N2	44 (13.2)	36 (12.6)	8 (17.0)	
**Metastases** *n (%)*				*0.187*
M0	280 (83.8)	237 (82.9)	42 (89.4)	
M1	54 (16.2)	49 (17.1)	5 (10.6)	
**R status** *n (%)*				*0.616*
R0	325 (97.3)	279 (97.6)	45 (95.7)	
R1	6 (1.8)	5 (1.7)	1 (2.1)	
RX	3 (0.9)	2 (0.7)	1 (2.1)	
**UICC stadium** *n (%)*				*0.709*
0	25 (7.5)	21 (7.3)	4 (8.5)	
I	93 (27.8)	81 (28.3)	12 (25.5)	
II	71 (21.3)	61 (21.3)	10 (21.3)	
III	91 (27.2)	75 (26.1)	16 (34.0)	
IV	54 (16.2)	49 (17.1)	5 (10.6)	
**Histological grading** *n (%)*				*0.708*
Undetermined	193 (57.8)	170 (59.4)	23 (48.9)	
Well differentiated	8 (2.4)	7 (6.0)	1 (4.2)	
Moderately differentiated	115 (34.4)	94 (80.3)	21 (87.5)	
Poorly differentiated	14 (4.2)	13 (11.1)	1 (4.2)	
Undifferentiated	4 (1.2)	3 (2.6)	1 (4.2)	

### Postoperative complications

Overall, 177 patients (52.9%) developed any postoperative complication. Data on postoperative complications are depicted in Table 4. Patients with AL were more likely to develop more severe complications as illustrated by Clavien Dindo Classification (CDC) (CDC ≥ IIIa: 83.0% vs. 23.1%; *p* < 0.001). Accordingly, these patients presented higher CCI (37.3 vs. 11.4; *p* < 0.001), higher rates of superficial surgical site infections (21.3% vs. 10.5%; *p* = 0.036) and longer LOS (22.3 vs. 12.5 days; *p* < 0.001). Perioperative 90-day mortality did not differ between groups (*p* = 0.366).

**Table 4:** Postoperative complications

	Total (*n* = 334)	No anastomotic leakage (*n* = 287)	Anastomotic leakage (*n* = 47)	*P* value
**CDC** *n (%)*				***< 0.001***
CDC I + II	108 (32.3)	100 (76.9)	8 (17.0)	
CDC ≥ IIIa	69 (20.7)	30 (23.1)	39 (83.0)	
**CCI** *avg (95%CI)*	15.1 (13.1–17.1)	11.4 (9.5–13.3)	37.3 (33.1–41.5)	***< 0.001***
**Ileus** *n (%)*	69 (20.7)	58 (20.2)	11 (23.4)	*0.370*
**Intraabdominal collection** *n (%)*	8 (2.4)	7 (2.4)	1 (2.1)	*0.687*
**Superficial surgical site infection** *n (%)*	40 (12.0)	30 (10.5)	10 (21.3)	***0.036***
**Pneumonia** *n (%)*	4 (1.2)	4 (1.4)	0 (0.0)	*0.544*
**Cardial complication** *n (%)*	3 (0.9)	2 (0.7)	1 (2.1)	*0.366*
**Thromboembolic complication** *n (%)*	2 (0.6)	1 (0.3)	1 (2.1)	*0.262*
**Urinary tract infection** *n (%)*	17 (5.1)	11 (3.8)	6 (12.8)	***0.021***
**Delirium** *n (%)*	8 (2.4)	6 (2.1)	2 (4.3)	*0.313*
**Central line infection** *n (%)*	4 (1.2)	4 (1.4)	0 (0.0)	*0.544*
**LOS** [d] *avg (95%CI)*	13.9 (13.1–14.6)	12.5 (11.9–13.1)	22.3 (19.2–25.3)	***< 0.001***
**Mortality 90 days** *n (%)*	3 (0.9)	2 (0.7)	1 (2.1)	*0.366*

CDC = Clavien Dindo Classification, CCI = Comprehensive Complication Index, LOS = Length of stay

### Details of AL management

Detailed description of AL assessment and management is shown in Table 5. All AL led to a change in the patients’ managements, which is derived from the Rahbari grading (Grade B: 53.2%; Grade C 46:8%) [36, 37]. AL was diagnosed after 22.3 days on average (95%CI 9.3–35.3 days). Analysis of the reported time points of AL diagnosis revealed two time frames. Therefore, a subgroup analysis was conducted comparing patients with AL diagnosis within 30 days of operation (early AL: 41/47 patients) to patients with leakage diagnosis after more than 30 days (late AL: 6/47 patients) after surgery. Patients with early AL received faster initiation of primary treatment compared to patients with late AL (0.2 vs. 5.3 days; *p* < 0.001).

**Table 5:** Description and management of anastomotic leakage

	Total (*n* = 47)	Early AL (*n* = 41)	Late AL (*n* = 6)	*P* value
**Time from surgery to diagnosis of AL** *[d] avg (95%CI)*	22.3 (9.3–35.3)	7.8 (5.8-9-9)	121.3 (53.1-189.6)	***< 0.001***
**Time from diagnosis of AL to treatment** [d] *avg (95%CI)*	0.8 (0.0-1.7)	0.15 (0.0-0.3)	5.3 (-2.8-13.5)	***< 0.001***
**Rahbari grading** ^**a**^ **of AL** *n (%)*				
Grade A	0 (0.0)	0 (0.0)	0 (0.0)	*0.603*
Grade B	25 (53.2)	22 (53.7)	3 (50.0)	
Grade C	22 (46.8)	19 (46.3)	3 (50.0)	
**AL related CDC** *n (%)*				*0.743*
AL CDC I + II	1 (2.1)	1 (2.4)	0 (0.0)	
AL CDC IIIa	18 (38.3)	16 (39.0)	2 (33.3)	
AL CDC IIIb	21 (44.7)	17 (41.5)	4 (66.7)	
AL CDC IVa	6 (12.8)	6 (14.6)	0 (0.0)	
AL CDC IVb	1 (2.1)	1 (2.4)	0 (0.0)	
AL CDC V	0 (0.0)	0 (0.0)	0 (0.0)	
**Primary treatment** *n (%)*				*0.558*
Non-surgical	26 (55.3)	23 (56.1)	3 (50.0)	
Antibiotic treatment	1 (3.8)	1 (4.3)	0 (0.0)	
Transanal catheter	15 (57.7)	14 (60.9)	1 (33.3)	
EVT	10 (38.5)	8 (34.8)	2 (66.7)	
Surgical	21 (44.7)	18 (43.9)	3 (50.0)	
**Success of primary therapy** *n (%)*				
Non-surgical	20/26 (76.9)	18/23 (78.3)	2/3 (66.7)	*0.562*
Antibiotic treatment	1/1 (100.0)	1/1 (100.0)	-	*-*
Transanal catheter	10/15 (66.7)	10/14 (71.4)	0/1 (0.0)	*0.333*
EVT	9/10 (90.0)	7/8 (87.5)	2/2 (100.0)	*0.800*
Surgical	11/21 (52.4)	9/18 (50.0)	2/3 (66.7)	*0.538*

^a^ Rahbari grading: Grade A = Anastomotic leakage results in no change in patient’s management, Grade B: Leakage requires active therapeutic intervention but is manageable without relaparotomy, Grade C: Anastomotic leakage requires relaparotomy

AL = anastomotic leakage, CDC = Clavien Dindo Classification, EVT = endoscopic vacuum therapy

The majority of patients received interventions with or without general anaesthesia (CDC IIIa: 38.3%, CDC IIIb: 44.7%) with no significant differences between patients with early or late AL diagnosis. Primary leakage treatment was grouped in NOLT and surgical management. NOLT included antibiotics, transanal catheter and EVT while surgical treatments included transabdominal or transanal reoperations under general anaesthesia. In the cohort of 26 patients (55.3%) with NOLT one patient received antibiotics only (3.8%), 15 patients received transanal catheter (57.7%) and 10 patients were treated with EVT (38.5%). 21 patients (44.7%) required primary reoperation. The indications for reoperation were the need for simultaneous ostomy creation (9 patients, 42.9%), sepsis (3 patients, 14.3%), clinical deterioration with peritonism and acute abdomen (7 patients, 33.3%) or unknown (2 patients, 9.5%). There were no statistically significant differences in therapy regimes of early and late AL (*p* = 0.556).

**Fig. 1:**
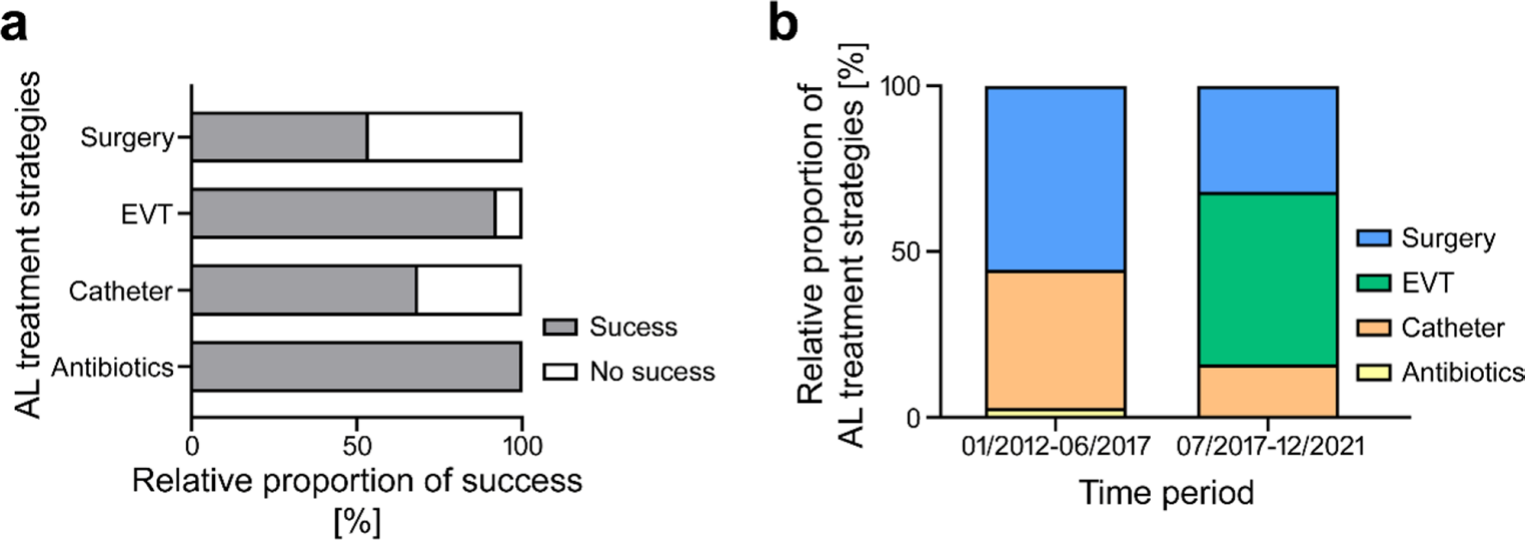
Success and strategies of AL therapy (**a**) Treatment success of different AL therapy strategies. (**b**) Proportions of different treatments strategies in separate time frames. AL = anastomotic leakage, EVT = endoscopic vacuum therapy

Overall success of primary AL therapy was achieved in 31/47 patients (66.0%). NOLT of AL showed a strong trend towards higher success rates than surgical management of AL (76.9 vs. 52.4%; *p* = 0.073) with no significant differences between early or late AL treatment. Within the NOLT options, EVT showed the highest treatment success rates with 90.0% (Fig. 1a).

When analysing the treatment strategies for AL in detail, it was observed that since EVT was implemented in 2017, the rates of surgical AL treatment decreased from 51.7 to 33.3% while NOLT of AL was increasingly used from 48.3 to 66.7% (*p* = 0.176) (Fig. 1b). Interestingly, the success rate of AL therapy slightly increased from 62.1 to 72.2% between these time periods (*p* = 0.349) with increasing success of NOLT (71.4% vs. 83.3%; *p* = 0.404) while success of surgical AL treatment remained unchanged (53.3% vs. 50.0%, *p* = 0.633).

Of the 6 patients with unsuccessful primary NOLT (5 patients with transanal catheter, one patient with EVT), 2 patients were successfully treated with EVT and 4 patients with surgery as second line treatment. Of the 10 patients with unsuccessful surgical AL treatment, 6 patients had received Hartmann resection, one patient needed a second reoperation, one patient was treated with transanal catheter and two patients received successful EVT. Overall, there were 13 patients that had been treated with EVT. Of these patients, all patients already had a protective ostomy. Description of EVT data are presented in Suppl. Table 1. Mean duration of EVT was 9.7 days with an average number of 1.6 sponge changes per case. No patient developed sepsis after start of EVT. EVT related complications were analgesic-sensitive pain in two cases (15.4%) and one case of post-EVT stricture (7.7%), which could be resolved with endoscopic dilatation.

### Oncological outcomes of patients with rectal cancer

To examine whether the type of AL management has an influence on oncological outcomes of rectal cancer patients with respect to adjuvant therapies and recurrence rates, an analysis of patients with early AL in patients with rectal cancer was performed and compared to patients without a leak.

**Fig. 2:**
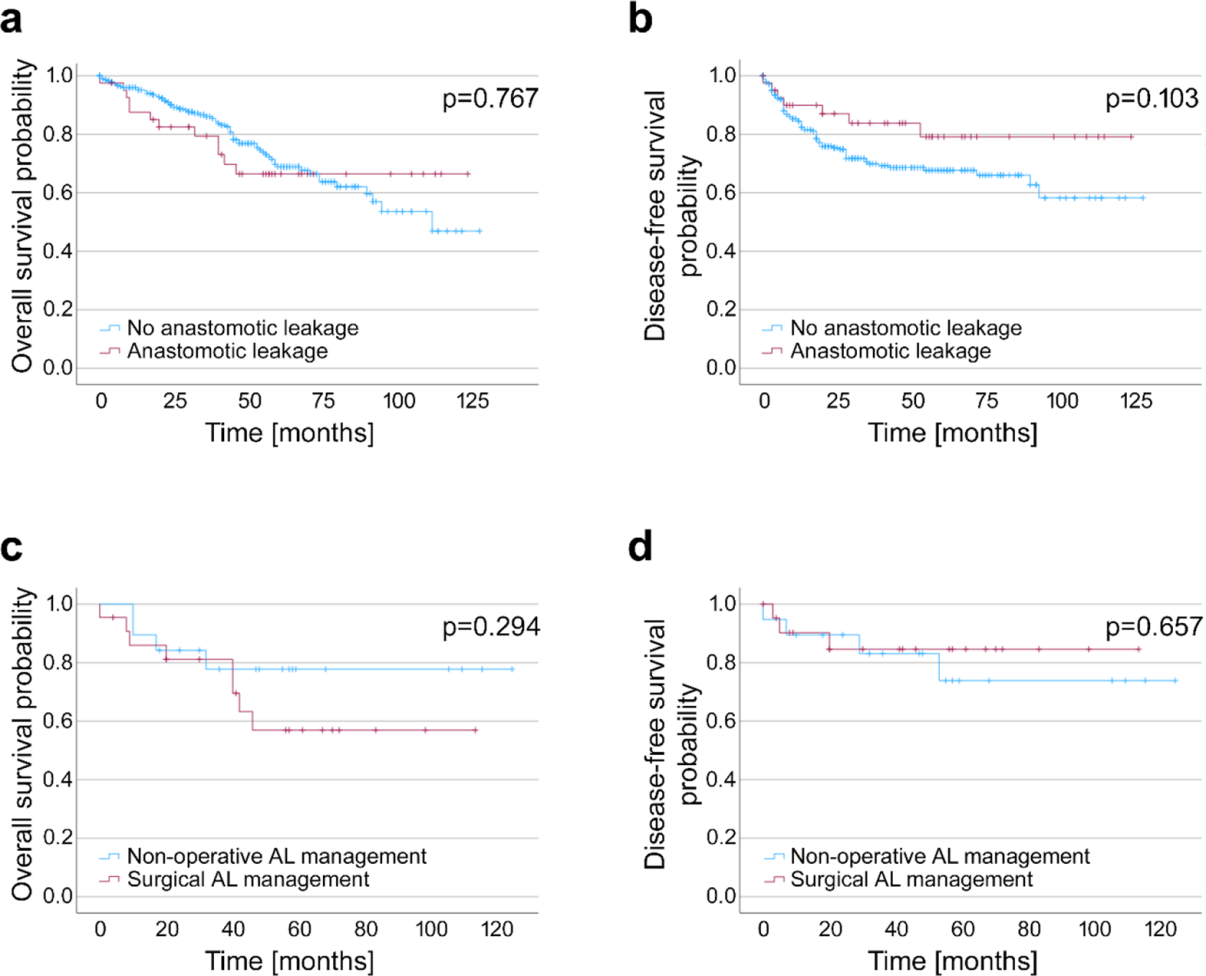
Overall and disease-free survival of patients with rectal cancer (**a**) OS of all patients with rectal cancer comparing patients with and without AL. (**b**) DFS of all patients with rectal cancer comparing patients with and without AL. (**c**) OS of patients with rectal cancer and early diagnosis of AL comparing non-surgical and surgical treatment. (**d**) DFS of patients with rectal cancer and early diagnosis of AL comparing non-surgical and surgical treatment. AL = anastomotic leakage, DFS = disease-free survival, OS = overall survival

Kaplan-Meier curves for OS and DFS are given in Fig. 2. Occurrence of AL had no significant effect on OS in our cohort with mean survival of 91.4 months in patients without a leak (95%CI [84.2; 98.6]) compared to 90.7 months in patients with AL (95%CI [75.1; 106.3]; *p* = 0.767). DFS in our cohort showed a trend towards better outcome in patients with AL, which did not reach statistical significance (no AL: 88.0 months, 95%CI [80.3; 95.7]; AL: 102.5 months, 95%CI [88.1; 116.9]; *p* = 0.103). When including the type of AL management, it was observed that NOLT resulted in a trend towards better mean survival (100.4 months, 95%CI [79.9; 120.9]) compared to reoperation (76.3 months; 96%CI [56.5; 96.0]; *p* = 0.294) missing statistical significance. DFS did not show significant differences (NOLT: 98.7 months, 95%CI [76.9; 120.4]; reoperation: 97.1, 95%CI [80.5; 113.7]; *p* = 0.657). Details on adjuvant therapy are depicted in Table 6. Adjuvant therapy was recommended in 56.6% of cases (189 patients) with no differences between patients with or without AL or type of AL treatment (*p* = 0.442). Overall, 88.4% of patients with recommended adjuvant therapy did receive it. When examining the subgroups there was a trend towards higher rates of executed adjuvant therapies in patients without AL (150 patients, 90.4%) compared to patients with AL (16 patients, 80.0%; *p* = 0.368). Although there was a trend towards higher rates of received adjuvant therapy after surgical AL treatment (10/10 patients vs. 6/10 patients, *p* = 0.082), this did not reach statistical significance due to the low number of patients. Interestingly, reasons for not receiving adjuvant therapy in the NOLT group were rejection by the patient (3/4 patients) and unfitness for therapy due to AL (1/4 patients). Occurrence of AL or type of AL treatment had no significant influence of the interval between surgery and beginning of adjuvant therapy.

**Table 6:** Adjuvant therapy in patients with rectal cancer

	Total (*n* = 334)	No AL (*n* = 287)	Early AL (*n* = 41)		*P* value
			Non-surgical management (*n* = 19)	Surgical management (*n* = 22)	
**Adjuvant therapy recommended** *n (%)*	189 (56.6)	166 (57.8)	10 (52.6)	10 (45.5)	*0.442*
**Adjuvant therapy received** *n (%)*	167 (88.4)	150 (90.4)	6 (60.0)	10 (100.0)	*0.082*
Data missing	5	4	1	0	
**Time from surgery to adjuvant therapy** [d] *avg (95%CI)*	48.2 (44.7–51.7)	47.3 (43.6–51.0)	55.6 (19.4–91.8)	56.6 (43.1–70.0)	*0.940*

LOS = length of stay

## Discussion

The aim of this study was to analyse the effects of different treatment strategies of AL after rectal resections for patients with rectal cancer, particularly with respect to oncological outcomes. Especially the increasing use of EVT as part of NOLT changes the scope of treatment modalities.

The AL rate of 14.1%, in our cohort was comparable to overall published AL rates after rectal resections [10–17, 19]. Univariate analysis revealed a number of patient-specific and procedure-specific risk factors for the development of AL. Most of them have been reported before to be associated with AL, such as male sex, high BMI, diabetes mellitus and anticoagulation [11, 14, 15, 17, 20, 21, 39, 40]. We observed leukaemia as a further risk factor for AL that had not been described before, although an incompetent immune system can understandably contribute to failure of anastomotic healing [40]. In our cohort, surgical approach did not influence the AL rate. Analysis of anastomotic technique on the other hand showed better healing rates for stapled anastomoses compared to partly or fully hand-sewn anastomoses. Although previous analyses did not report differences between surgical techniques for colorectal resections [41], there have been a few reports on superior anastomotic healing after stapled technique in ileocolic anastomoses [40, 42]. Interestingly, if no test for leakage was performed, there was a higher risk for developing an AL, suggesting that a defective test led to imminent therapeutic consequences. However, oversewn anastomoses still presented higher insufficiency rates in our cohort. This emphasizes the importance of achieving a primarily sufficient anastomosis during surgery, while mended anastomoses seem to heal inferiorly. Protective ostomy and pelvic drainage did not impact the AL rate though some authors have reported a benefit of protective ostomy for the development of AL [11, 12].

With regard to the patients with rectal cancer, we did not identify any preoperative tumour characteristics to influence AL rate. Although advanced tumour stage has been reported to increase the risk of AL [15, 40, 42], we did not observe similar trends in our cohort. Furthermore, neoadjuvant therapy did not increase AL rate in our patients while reports on postoperative AL rates after neoadjuvant therapy show conflicting results [14, 40, 42].

As expected, patients with AL presented significantly higher CCI and longer LOS. Furthermore, there were more superficial SSI in patients with AL. Although several studies have demonstrated that AL is associated with higher perioperative mortality [10, 11, 13, 14, 16, 39], we cannot confirm this according to our data. This could be related to the low overall perioperative mortality of 0.9% and AL associated mortality of 2.1% in our cohort, which lies below several reports by other study groups that observed AL related perioperative mortality between 1.3 and 18.3% [10, 11, 13, 15, 16, 39].

Analysing time frames of AL detection revealed two subgroups, early and late AL. While early AL became clinically evident after approximately 7.8 days after surgery, late AL represents the initially clinically unapparent AL that was diagnosed in routine examinations before ostomy reversal and did not cause apparent clinical impairment of the patients. The standard therapeutic measurements for AL after rectal resections used to consist of antibiotics, drainage or reoperation [16, 17, 22]. With EVT, there is a relatively new and very efficient therapy for AL treatment that expands the repertoire of non-operative AL management. In our cohort, AL treatment was investigated comparing NOLT and surgical therapies with equivalent distribution between the two therapy regimens. NOLT showed higher success rates compared to surgical AL treatment. While this difference did not reach statistical significance — most likely due to the limited sample size of patients with AL and resulting statistical underpowering — it remains clinically meaningful. Although a selection bias for the patients that require reoperation as first-line AL treatment is probable, an investigation of two time frames showed interesting results. While NOLT of AL was performed in 48.3% of patients between 2012 and 2017, implementing EVT in 2017 raised the rate of NOLT to 66.7%. This development suggests that a portion of patients that had previously been subjected to reoperation can nowadays be treated successfully with EVT, particularly given the improved success rates of NOLT in the later time period. To exclude any selection bias, a prospective study design would be needed with explicit treatment algorithms for AL, which was not at our disposal in this retrospective analysis. Duration of EVT was 9.7 days with 1.6 sponge changes per patient on average. Our treatment was shorter than reported EVT treatments with durations between 18 and 23 days and 5–8 sponge changes [19, 27, 28]. Still, EVT showed the highest success rate of 90.0% among all treatment strategies. This is comparable to previous reports on successful AL treatment with EVT after colorectal surgery [19, 24, 27, 28]. Even for late diagnoses of AL EVT showed similar treatment success. Although we acknowledge that there were only two patients with late AL that received EVT, our data suggests that EVT is also a viable option for treatment of late AL while reports by other authors observed higher success rates in early AL treatment [29, 30]. Complications of EVT were limited to analgesic sensitive pain and strictures that were successfully managed endoscopically. Other authors have reported similar complications after EVT [19, 23, 24].

One of the most interesting questions concerning tumour patients is whether AL has long-term effects on the oncological outcome. Although locally advanced rectal cancer is mostly treated with neoadjuvant chemoradiotherapy according to the national guidelines, we did not observe any effect of neoadjuvant treatment on AL rates. Therefore, intra- and postoperative parameters need to be examined when analysing diverging long-term outcomes of oncological patients. When examining the long-term follow up data, we observed no significant differences in OS between patients with or without AL. However, there seems to be a trend towards lower 5-year survival in patients with AL compared to patients without AL. These findings are in line with the observations of other study groups that AL is associated with shorter OS and lower 5-year survival rates [11, 20, 31, 32, 34, 43]. It has been discussed that this might be owed to fewer or delayed adjuvant therapies [20, 43]. Our data also shows a trend towards lower rates of executed adjuvant therapies in patients with AL although statistical significance was not reached, suggesting that prolonged recovery might interfere with oncologic treatment strategies. However, in our survival analysis the difference between cohorts was not statistically significant and trends redeemed after 5 years.

Interestingly, a subgroup analysis of different AL treatment strategies revealed that patients with NOLT of AL showed a trend towards better OS although adjuvant therapy was less likely to be performed than in patients with surgical AL therapy. This challenges the adjuvant therapy being the relevant criterion for OS in tumour patients with AL. Furthermore, we observed a trend towards better DFS in patients with AL, suggesting that the oncological characteristics might not be the cause of the lower OS. This might be due to the patient specific risk profile that predisposes them for AL and also causes non-tumour related mortality. Contrary to our data, some authors have reported shorter DFS and lower 5-year DFS rates for patients with AL [33, 34]. All in all, it is still widely discussed whether AL effects tumour progression. While some study results showed higher rates of local recurrence in patients with AL [20, 31, 33, 39], other groups did not support these findings [11, 34]. Similarly, distant tumour recurrence has been reported to be increased after AL [20, 32] while other authors did not observe a change in distant tumour recurrences [11, 31]. This demonstrates how controversially this topic is being discussed and how important it is to generate data in this field.

### Limitations

The main limitations of this study are the retrospective nature and small cohort of patients with AL as well as subgroups of different treatment strategies. Data were collected at a single centre. Due to the retrospective design, there were no standardised parameters for the decision-making process in AL treatment. Also, over the decade rectal resection changed towards minimally-invasive surgery, especially robotic surgery led to a reduction of LOS and overall complications [44]. The implementation of EVT changed the tendencies towards conservative AL management over the timespan of the study [26]. Furthermore, the criteria for sponge changes, termination of EVT were decided by the clinical impression of the endoscopist, which are of subjective nature. In our belief, a prospective study with an exact protocol for planned postoperative endoscopic control for AL, leakage management regimen including intervals of sponge changes and ending of leakage therapy is necessary.

## Conclusion

Our study confirms that EVT enables NOLT of AL after rectal resection and constitutes a safe and successful therapy strategy. Development of AL seems to coincide with lower 5-year survival in patients with rectal cancer which is not associated with tumour progression but might be rather due to predisposing patient characteristics. NOLT of AL has the potential of increasing long-term OS of patients with rectal cancer. However, further studies are necessary to investigate the efficacy and influence of EVT in rectal resection leakage therapy.

## Electronic supplementary material

Below is the link to the electronic supplementary material.

Supplementary Material 1

## Data Availability

No datasets were generated or analysed during the current study.
