# Supplementary material for: The effects of endoscopic vacuum therapy for non-operative treatment of anastomotic leakage on oncological outcomes in rectal cancer patients
Source: Langenbecks Arch Surg. 2025 Mar 27;410(1):107. doi: 10.1007/s00423-025-03672-1 (PMC11950071; doi:10.1007/s00423-025-03672-1)
Supplement: Supplementary file 1 — Supplementary Material 1 [file 423_2025_3672_MOESM1_ESM.docx]

Supplementary Table

Supplementary Table 1: Description of EVT

|  | Total  (n=13) |  |
| --- | --- | --- |
|  |  |  |
| **Success of EVT** *n (%)* | 12 (92.3) |  |
| **Duration of EVT** [d] *avg (95%CI)* | 9.7 (6.7-12.7) |  |
| **Number of sponge changes** *avg (95%CI)* | 1.6 (0.9-2.3) |  |
| **New onset of sepsis during EVT** *n (%)* | 0 (0.0) |  |
| **Complications of EVT** *n (%)* |  |  |
| Bleeding | 0 (0.0) |  |
| Pain | 2 (15.4) |  |
| Stricture | 1 (7.7) |  |

EVT=endoscopic vacuum therapy
